# S100A8/A9 (Calprotectin) Negatively Regulates G2/M Cell Cycle Progression and Growth of Squamous Cell Carcinoma

**DOI:** 10.1371/journal.pone.0069395

**Published:** 2013-07-09

**Authors:** Ali Khammanivong, Chengxing Wang, Brent S. Sorenson, Karen F. Ross, Mark C. Herzberg

**Affiliations:** 1 Department of Diagnostic and Biological Sciences, University of Minnesota, Minneapolis, Minnesota, United States of America; 2 Mucosal and Vaccine Research Center, University of Minnesota, Minneapolis, Minnesota, United States of America; Bambino Gesu' Children Hospital, Italy

## Abstract

Malignant transformation results in abnormal cell cycle regulation and uncontrolled growth in head and neck squamous cell carcinoma (HNSCC) and other cancers. S100A8/A9 (calprotectin) is a calcium-binding heterodimeric protein complex implicated in cell cycle regulation, but the specific mechanism and role in cell cycle control and carcinoma growth are not well understood. In HNSCC, S100A8/A9 is downregulated at both mRNA and protein levels. We now report that downregulation of S100A8/A9 correlates strongly with a loss of cell cycle control and increased growth of carcinoma cells. To show its role in carcinogenesis in an in vitro model, S100A8/A9 was stably expressed in an S100A8/A9-negative human carcinoma cell line (KB cells, HeLa-like). S100A8/A9 expression increases PP2A phosphatase activity and p-Chk1 (Ser345) phosphorylation, which appears to signal inhibitory phosphorylation of mitotic p-Cdc25C (Ser216) and p-Cdc2 (Thr14/Tyr15) to inactivate the G2/M Cdc2/cyclin B1 complex. Cyclin B1 expression then downregulates and the cell cycle arrests at the G2/M checkpoint, reducing cell division. As expected, S100A8/A9-expressing cells show both decreased anchorage-dependent and -independent growth and mitotic progression. Using shRNA, silencing of S100A8/A9 expression in the TR146 human HNSCC cell line increases growth and survival and reduces Cdc2 inhibitory phosphorylation at Thr14/Tyr15. The level of S100A8/A9 endogenous expression correlates strongly with the reduced p-Cdc2 (Thr14/Tyr14) level in HNSCC cell lines, SCC-58, OSCC-3 and UMSCC-17B. S100A8/A9-mediated control of the G2/M cell cycle checkpoint is, therefore, a likely suppressive mechanism in human squamous cell carcinomas and may suggest new therapeutic approaches.

## Introduction

Loss of growth suppression is one of the hallmarks of cancer [1], contributing to malignant transformation and tumorigenesis. The molecular mechanisms leading to abnormal cell cycle regulation and growth vary in different types of cancer and remain elusive in head and neck squamous cell carcinoma (HNSCC). Calprotectin or S100A8/A9, a heterodimeric complex of calcium-binding proteins S100A8 (MRP8 or calgranulin A) and S100A9 (MRP14 or calgranulin B), may play a role in growth regulation and tumorigenesis in HNSCC and other squamous cell carcinomas (SCCs). S100A8/A9 is constitutively expressed in the cytoplasm of healthy squamous epithelial cells of the oral cavity and oropharynx [2], [3]. Encoded by genes that map to human epithelial differentiation complex on chromosomal locus 1q21, S100A8 and S100A9 are members of the S100 family of proteins, which contain two canonical EF-hand calcium-binding motifs involved in calcium-dependent control of cell differentiation, cell cycle progression and growth [4] and are implicated in cancer development and other inflammatory diseases. In cancer, extracellular S100A8/A9, typically released from the cytoplasm of infiltrating polymorphonuclear leukocytes and macrophages [5], [6], is associated with inflammation and progression of the disease [7]. When released, S100A8/A9 can signal through the receptor for advanced glycation end products (RAGE) and toll-like receptor 4 (TLR4) to promote tumor-associated inflammation and progression of advanced stage adenocarcinomas and colitis-associated cancer [8]–[11]. Little is known, however, about the intracellular roles of S100A8/A9 and how the protein complex regulates biological functions in cancer cells.

Expression of S100A8/A9 appears to be cell- and tissue-specific and is differentially regulated in different malignancies. S100A8/A9 levels are usually abnormally elevated in human primary tumors originating from tissues that do not normally express the protein, such as the skin [7], breast [12], [13], thyroid [14], liver [15], gastric mucosa [16], prostate [8], ovary [17], bladder [18] and lung [19]. In these tissues, whether increased S100A8/A9 level is a response to tumorigenesis or actually drives tumor development and progression is unclear. In contrast, S100A8/A9 expression decreases in human tumors of squamous epithelial cell origin that normally express the protein complex constitutively, such as head and neck (including oral, nasopharyngeal and oropharyngeal) [20]–[22], esophageal [23], [24] and cervical [25], [26] SCCs. In these squamous epithelial cancers, decreased S100A8/A9 is highly correlated with loss of differentiation and increase in growth and invasiveness. Conversely, S100A8/A9-expressing SCCs appear less aggressive. We sought to determine, therefore, whether S100A8/A9 functions as a growth regulating factor in SCCs.

To test the regulatory role of S100A8/A9 by rescue in S100A8/A9-negative carcinoma cells, we stably transfected KB cells to express S100A8/A9 protein complex. We now show that stable expression of S100A8/A9 in KB cells results in S100A8/A9-dependent G2/M cell cycle arrest and reduced anchorage-dependent growth and colony formation in soft agar. To determine the effect of reducing S100A8/A9 levels, S100A8 and S100A9 were silenced in the TR146 cells using short hairpin RNA (shRNA). In TR146 cells, silencing of S100A8 and S100A9 expression reverses the suppressive effect on growth and clonogenicity and appears to be associated with the loss of G2/M checkpoint control. Both G1/S and G2/M cell cycle checkpoints are critical in regulating normal cell division and growth, but are dysregulated in carcinomas leading to uncontrolled growth and proliferation. Growth regulation by S100A8/A9 in KB cells was not found to be through G1, G1/S checkpoint or S phase. Instead, S100A8/A9 expression increases protein phosphatase 2A (PP2A) activity, apparently through protein-protein interaction, which appears to be essential in modulating and restoring G2/M checkpoint signaling and reduction of carcinoma growth.

PP2A is a serine and threonine (Ser/Thr) protein phosphatase known to regulate cell cycle checkpoint by targeting G2/M-specific Cdc25C for inactivation by dephosphorylation at Thr48, inhibiting the mitotic exit and cell division [27], [28]. PP2A targets a broad spectrum of phosphoproteins and has also been shown to exert anti-tumor activities by inhibiting AKT and C-MYC in the cell survival and proliferation pathways and by inducing cell cycle arrest [29]. When interacting with S100A8/A9 [30], PP2A may be activated to exert regulatory roles in cell cycle progression at the G2/M checkpoint. S100A8/A9 and PP2A may therefore function as interacting partners to regulate mitosis and control cellular growth. Reduction in S100A8/A9 in carcinomas (in HNSSC for example) may lead to a diminished PP2A phosphatase activity and increased growth and tumorigenesis.

## Results

### S100A8 and S100A9 gene expression by qRT-PCR in normal and SCC tissues

**Figure 1:**
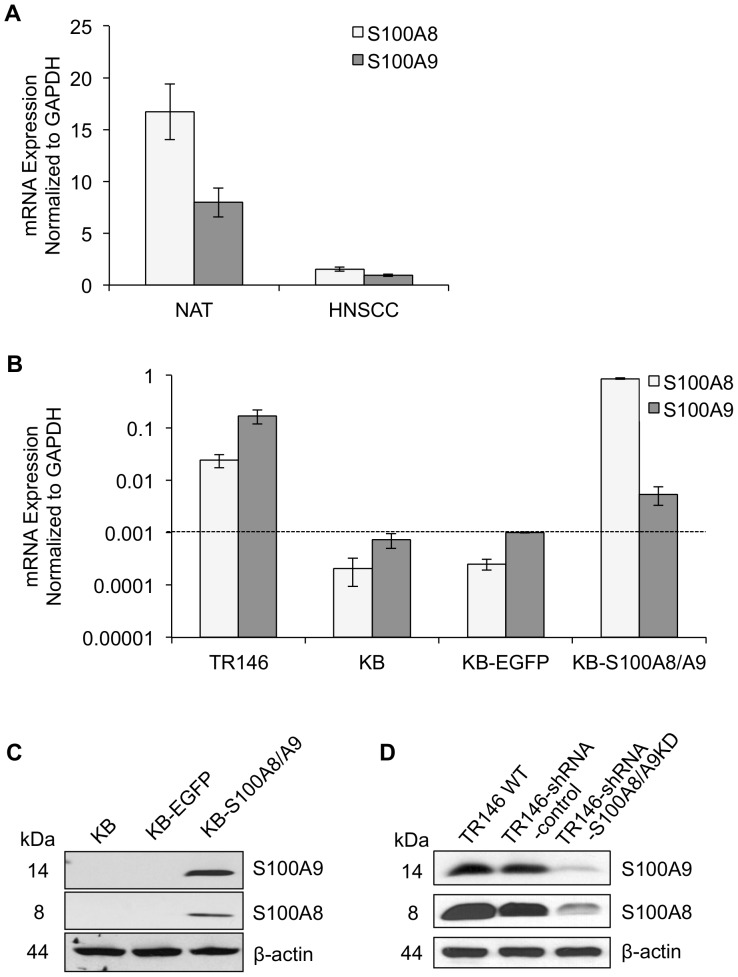
Expression of S100A8 and S100A9 in human head and neck tissues and carcinoma cell lines. The mRNA expression levels of S100A8 and S100A9 in (A) normal adjacent (NAT) and HNSCC tissues and (B) TR146 HNSCC and KB cells were measured by qRT-PCR and normalized to GAPDH; dotted line shown as a threshold for detection. Total RNA extracted from matching NAT and HNSCC tissues from each of three patients was pooled separately for gene expression analysis. Cell lines cultured under standard conditions were harvested and analyzed at approximately 70% confluency. Data presented as mean ±SD (n = 2). Representative immunoblots of (C) S100A8 and S100A9 in KB-S100A8/A9 transfected cells and (D) TR146-shRNA-S100A8/A9 knockdown cells compared to wild type and negative transfection controls. β-actin was used as loading control for immunoblotting analysis separated in 10% SDS-PAGE gels.

S100A8/A9 protein expression is reduced in HNSCC as reported previously [31], [32]. In this study, we compared the expression of *S100A8* and *S100A9* subunit genes in matching HNSCC and normal adjacent (NAT) tissues using real-time quantitative reverse-transcription polymerase chain reaction (qRT-PCR). Expression analysis was normalized to GAPDH housekeeping gene as an internal and total RNA loading control. Total RNA from HNSCC and NAT tissues were pooled separately from three patients with stage II, III and IVA tumors and analyzed for mRNA expression using qRT-PCR. We found that S100A8 and S100A9 expression was approximately 10-fold lower in HNSCC than NAT, the normal tissue control (Figure 1A). Using microarray gene expression profiling, we have also investigated the level of S100A8 and S100A9 expression in over 35 clinical cases of HNSCC and eleven healthy oral mucosal samples, which confirm the qRT-PCR results (data not shown; manuscript submitted for publication).

Expression of S100A8 and S100A9 in TR146 and KB-S100A8/A9 cells was approximately one- to two-log-fold greater than in S100A8/A9-negative KB and KB-EGFP cells (Figure 1B; dotted line indicates threshold of detection). KB-S100A8/A9 cells are S100A8/A9-negative KB cells transfected to over-express S100A8/A9, whereas KB-EGFP cells are sham-transfected controls. In KB wild-type and sham-transfected KB-EGFP cells, S100A8 and S100A9 mRNA and protein expression was barely detectable, whereas transfected KB-S100A8/A9 and TR146 cells clearly showed S100A8 and S100A9 protein expression (Figure 1C, D). S100A8- and S100A9-specific stable shRNA transfection of TR146 cells reduced endogenous S100A8 and S100A9 proteins to barely detectable levels (Figure 1D). Note that S100A8 and S100A9 protein levels appear to differ as shown by immunoblotting (Figure 1C, D). Such differences may be real or reflect differences in the sensitivity of the primary antibodies used. Hence the levels of S100A8 and S100A9 could not be directly compared to one another.

### Anchorage-dependent and -independent growth of carcinoma cells suppressed by S100A8/A9

**Figure 2:**
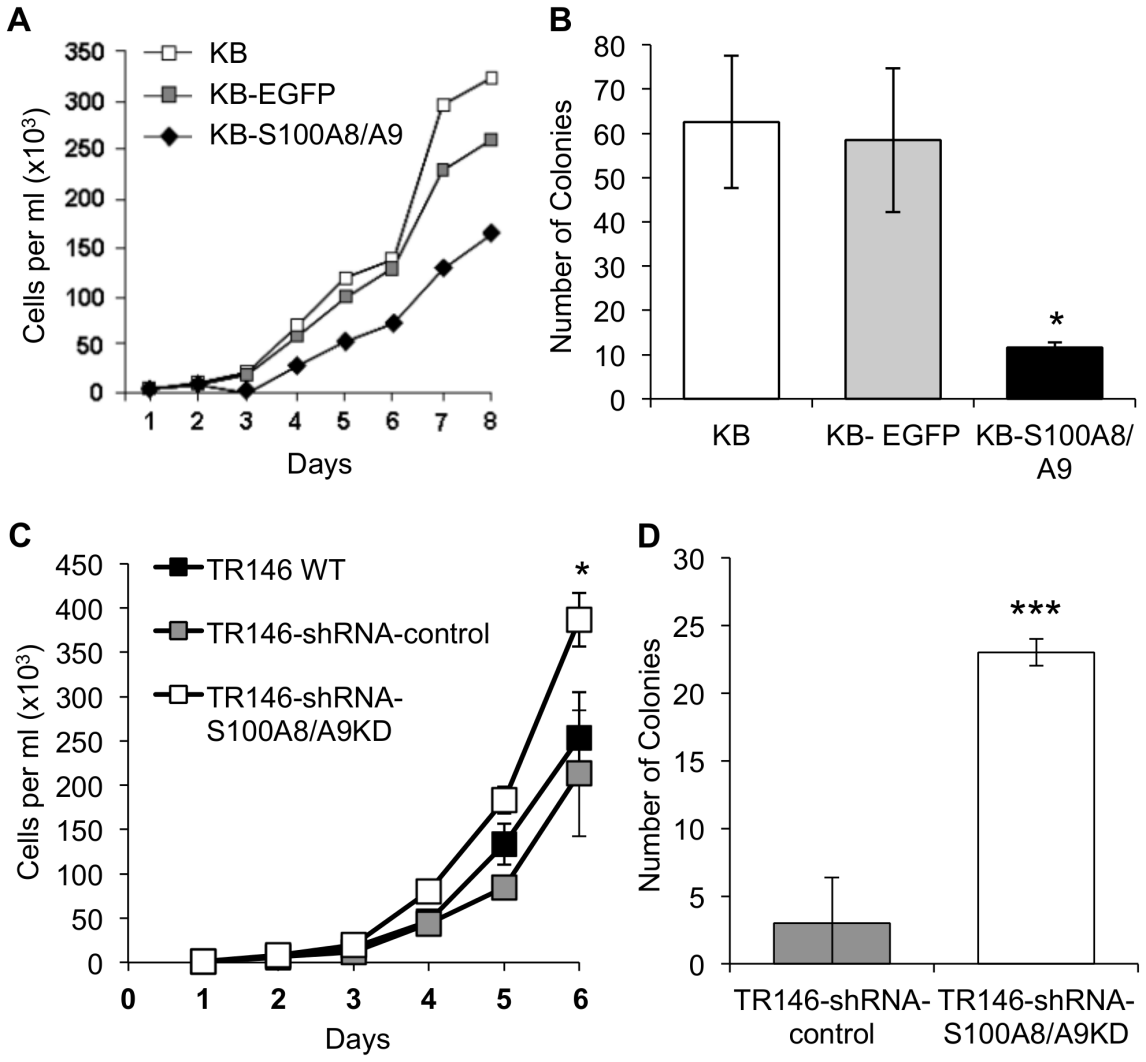
S100A8/A9 suppressed anchorage-dependent and -independent growth of KB cells. (A) Growth curves of KB-S100A8/A9 cells compared to KB-EGFP and KB wild-type control cells. Cells were grown on non-pyrogenic polystyrene tissue flasks in fresh MEM supplemented with 10% FBS every three days. (B) Anchorage-independent growth in soft agar for KB, KB-EGFP and KB-S100A8/A9 cells (mean ±SEM, *p<0.03, n = 4). (C) Anchorage-dependent growth on tissue flasks in complete medium (mean ±SD, *p<0.02, n = 3) and (D) colony formation in soft agar (mean ±SEM, ***p<0.0005, n = 2) of TR146-shRNA-S100A8/A9KD cells compared to TR146 WT and TR146-shRNA-control cells.

The ability of S100A8/A9 to regulate cell growth was tested in transfected KB-S100A8/A9 cells on standard non-pyrogenic polystyrene tissue culture flasks for anchorage-dependent (adherent) and in soft agar for anchorage-independent growth conditions. Highly malignant cells are able to survive, form colonies and proliferate independent of anchorage and are routinely tested in soft agar. When compared to KB wild-type or KB-EGFP cells, KB-S100A8/A9 cells showed approximately two-fold lower anchorage-dependent growth in vitro (Figure 2A). Ectopic expression of S100A8/A9 did not appear to induce apoptosis in KB-S100A8/A9 cells when grown under standard adherent conditions, since cleaved caspases 1 and 3 were not detectable by immunoblot, whereas KB wild-type, KB-EGFP and KB-S100A8/A9 cells showed similar total caspase 1 and 3 levels (data not shown). During anchorage-independent growth in soft agar, KB-S100A8/A9 cells grew poorly, forming smaller and fewer colonies than KB and KB-EGFP cells (Figure 2B). To confirm the effect of S100A8/A9 on cell growth, knockdown of endogenous S100A8/A9 expression in TR146-shRNA-S100A8/A9KD cells showed increased anchorage-dependent (Figure 2C) and -independent (Figure 2D) growth in comparison to knockdown-negative control cells (TR146-shRNA-control).

### S100A8/A9 induces G2/M cell cycle checkpoint arrest

**Figure 3:**
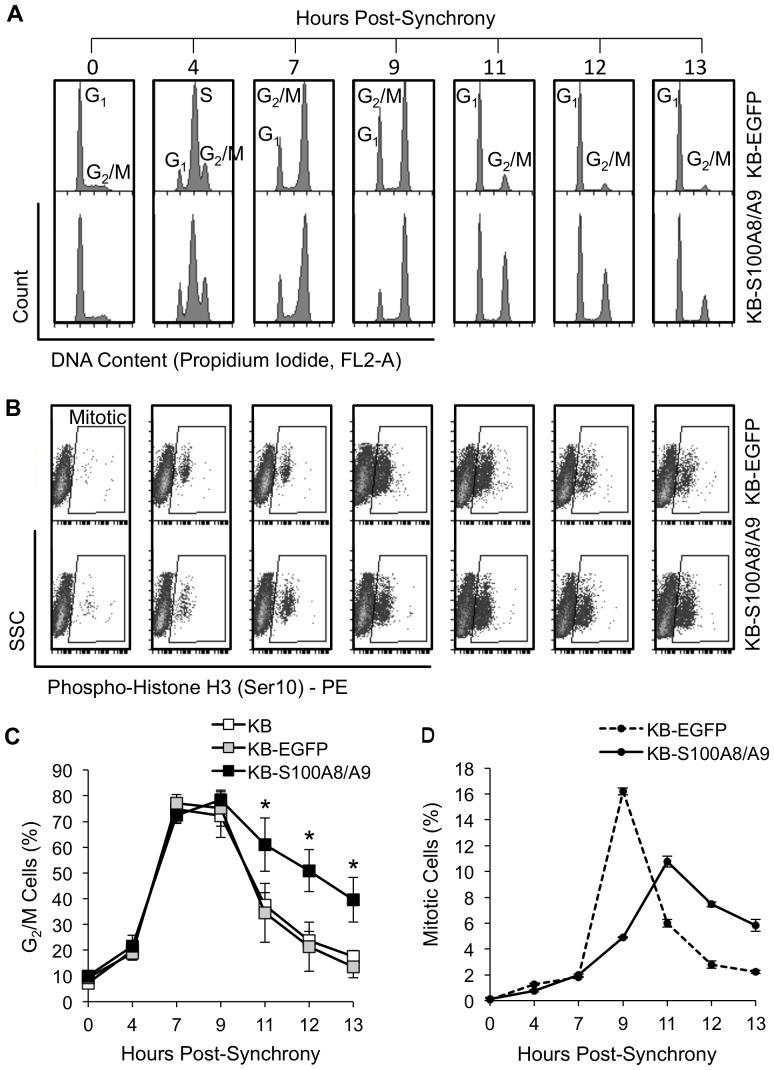
S100A8/A9 expression induced cell cycle and mitotic arrest at G2/M. (A) Cell cycle analysis of KB, KB-EGFP and KB-S100A8/A9 cells post-synchrony. Cells cultured under standard conditions were serum-starved overnight, synchronized at G1/S with aphidicolin treatment and stimulated to re-enter cell cycle. Synchronized cells were stained with propidium iodide DNA staining solution and analyzed by flow cytometry for changes in DNA content following release from G1/S blockage. (B) Mitotic analysis of synchronized cells stained with phospho-Histone H3 (Ser10) and analyzed by flow cytometry. (C) Percentage of cells in G_2_/M. KB, KB-EGFP and KB-S100A8/A9 cells were analyzed over time post-synchrony and reported as mean ±SEM; n = 2 independent experiments (each analysis performed in duplicate); *p<0.05. (D) Percentage of mitotic cells post-synchrony, representing the mean of two independent repeat experiments. KB-S100A8/A9 cells showed fewer mitotic cells as shown by lower phospho-Histone H3 (Ser10) staining.

To determine whether S100A8/A9 suppressed carcinoma cell growth by regulating cell cycle, we performed cell cycle analysis in S100A8/A9-expressing and non-expressing KB cells. Rapidly dividing cells such as carcinomas progress through the cell cycle at higher rates than normal cells. To determine differences in cell cycle progression, carcinoma cells were synchronized at G1/S phase by serum starvation overnight followed by treatment with aphidicolin for 12 h in normal growth medium. Following release from aphidicolin-induced G1/S blockage, KB wild-type, KB-EGFP and KB-S100A8/A9 cells progressed through G1/S phase and entered G2/M phase at approximately the same rate as determined by PI-staining of DNA (Figure 3A, C). KB and KB-EGFP cells exited G2/M phase by 11 hours and the G2/M cell population returned to background levels by 13 hours post-synchrony. KB-S100A8/A9 cells, however, appeared to arrest in G2/M phase for at least 3 hours. Consistent with these observations, KB-S100A8/A9 cells showed fewer mitotic cells at 9 hours post-synchrony and remained in metaphase longer than KB-EGFP cells (Figure 3B, D).

### S100A8/A9 modulates G2/M signaling pathway

**Figure 4:**
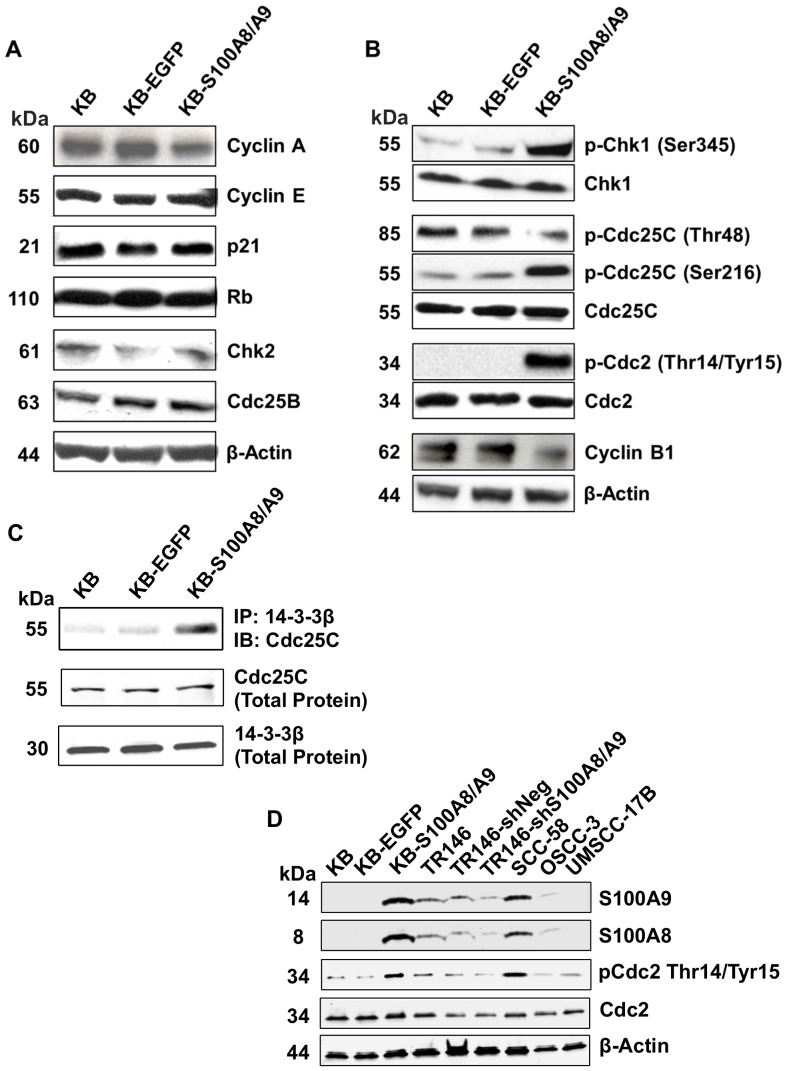
S100A8/A9 modulates G_2_/M cell cycle checkpoint regulating molecules in SCC. Expression and phosphorylation (activation/inactivation) status of cell cycle regulators are shown by immunoblotting analysis of G2/M synchronized cells. Data shown are representative of multiple independent repeat experiments. Goat anti-β-actin polyclonal IgG was used to detect β-actin as protein loading control. (A) Expression of G1/S regulating proteins, cyclin A, cyclin E, p21 and Rb, Chk2 and Cdc25B, in KB, KB-EGFP and KB-S100A8/A9. Phosphorylation of p-Chk2 (Thr68) was not detectable in any cell, with or without S100A8/A9 expression (not shown). (B) Expression and phosphorylation status of G2/M regulators, Chk1/p-Chk1 (Ser345), mitotic active p-Cdc25C (Thr48), Cdc25C/p-Cdc25C (Ser216), Cdc2/p-Cdc2 (Thr14/Tyr15), and cyclin B1. (C) Immunoblotting (IB) of Cdc25C protein co-immunoprecipitated (IP) with 14-3-3β captured with rabbit anti-14-3-3β polyclonal IgG. Immunoblotting of total 14-3-3β and Cdc25C proteins are also shown. (D) Protein levels of S100A8, S100A9, Cdc2 and p-Cdc2-(Thr14/Tyr15) in wild-type TR146 (TR146 WT), control shRNA transfectant (TR146-shRNA-control) and shRNA-induced S100A8/A9 knockdown (TR146-shRNA-S100A8/A9KD) cells relative to the levels in KB cells. Protein levels are also shown in several other HNSCC cell lines, SCC-58, OSCC-3 and UMSCC-17B with different levels of S100A8/A9 expression.

To confirm whether S100A8/A9 regulates the G2/M checkpoint, we tested protein expression and activation status of G1/S and G2/M cell cycle checkpoint regulators by immunoblotting of protein samples from cells synchronized at G2/M by nocodazole treatment. G2/M synchronized KB, KB-EGFP and KB-S100A8/A9 cells expressed similar levels of the cell cycle regulators cyclin A, cyclin E, p21, Rb, Chk2, and Cdc25B (Figure 4A), suggesting that the G1/S checkpoint was unaffected by S100A8/A9 expression. Cyclin B1, which is a G2/M checkpoint regulator required for cyclin-dependent kinase 1 (Cdc2) activities during entry into mitosis [33], showed lower mRNA (data not shown) and protein (Figure 4B) levels in KB-S100A8/A9 than KB and KB-EGFP cells. More significantly, S100A8/A9 expression increased phosphorylation of the G2/M-associated checkpoint kinase p-Chk1 (Ser345), phosphatase p-Cdc25C (Ser216) and p-Cdc2 (Thr14/Tyr15) in KB-S100A8/A9, but did not alter levels of these proteins (Figure 4B). S100A8/A9 also decreased the mitotic active form of p-Cdc25C (Thr48) (Figure 4B). In KB-S100A8/A9 cells, we found greater co-immunoprecipitation of Cdc25C with 14-3-3β (Figure 4C), suggesting increased interaction between Cdc25C and 14-3-3β. Total 14-3-3β protein expression was unaffected by S100A8/A9 expression. As expected, knockdown of S100A8/A9 in TR146-shRNA-S100A8/A9KD cells decreased p-Cdc2 (Thr14/Tyr15) phosphorylation (Figure 4D).

To show that S100A8/A9 levels generally correlated with hyperphosphorylation of p-Cdc2 (Thr14/Tyr15) in HNSCC, we tested three additional HNSCC cell lines with varying endogenous S100A8/A9 expression levels. When synchronized at G2/M, SCC-58 cells (high S100A8/A9 protein expression) showed strong hyperphosphorylation of p-Cdc2 (Thr14/Tyr15), but phosphorylation was nearly undetectable in OSCC-3 (low S100A8/A9 protein expression) and UMSCC-17B (undetectable S100A8/A9 protein expression) cells (Figure 4D). Taken together, our results suggest that S100A8/A9 modulates the canonical G2/M signaling pathway, resulting in inactivation of Cdc2 and maintenance of a G2/M checkpoint delay.

### S100A8/A9 interacts with PP2A phosphatase and increases activity

**Figure 5:**
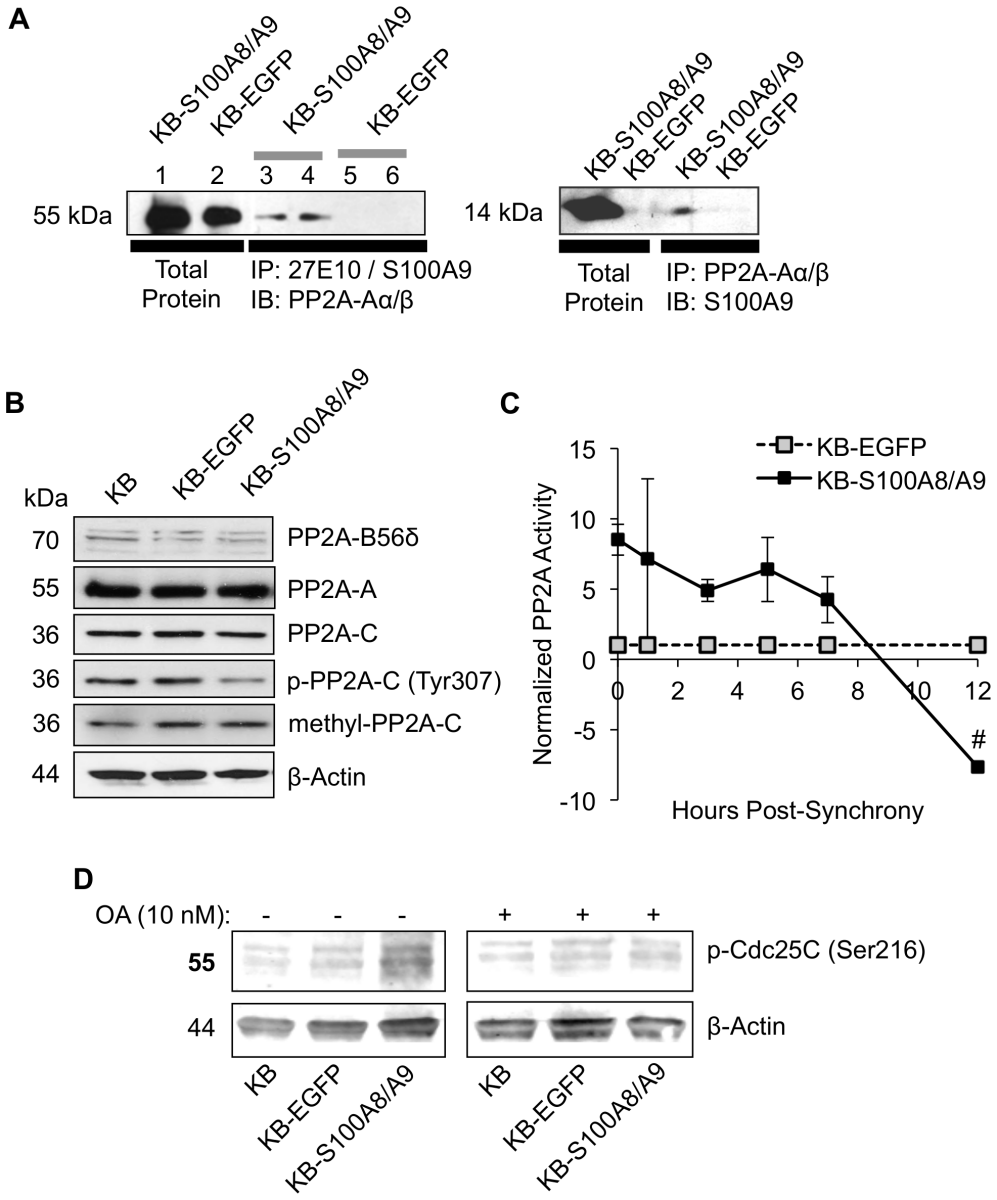
S100A8/A9 interaction with PP2A phosphatase increases activity. (A) PP2A-Aα/β co-immunoprecipitated (IP) with either S100A8/A9 complex (IP with 27E10 antibody) or S100A9 subunit (left panel) and S100A9 (used as S100A8/A9 complex marker protein) co-IP with PP2A-Aα/β subunit as detected by immunoblotting (IB). (B) PP2A subunit protein expression, phosphorylation and methylation compared in KB, KB-EGFP and KB-S100A8/A9 cells (synchronized at 5 h post-G1/S) as detected using IB. Blot shown is a representative of multiple repeats (n≥3) of total protein lysates separated in 10% SDS-PAGE gels. β-actin was used as protein loading control. (C) The PP2A-C phosphatase activities in KB-EGFP and KB-S100A8/A9 cells were normalized to the detectable PP2A-Aα/β co-immunoprecipitated with S100A8/A9 (27E10 antibody) shown in A, and then further normalized to KB-EGFP levels. Data was shown as Mean ±SE (n = 2). ^#^Error bars not visible. (D) Inhibition of S100A8/A9-dependent p-Cdc25C (Ser216) phosphorylation by 10 nM okadaic acid (OA). Expression of p-Cdc25C in the absence (left panel) and presence of OA (right) as detected by IB.

To identify candidate cell cycle regulators that might directly interact with S100A8/A9, a curated database of known and predicted protein-protein interacting partners (Ingenuity Pathway Analysis, Genedata Inc., San Francisco, CA USA) was interrogated. We found that S100A8/A9 might be a binding partner and modulator of activity of protein phosphatase 2A (PP2A), a known cell cycle regulator. PP2A is a heterotrimeric holoenzyme with three subunits: structural protein subunit A, regulatory subunit B, and catalytic subunit C. PP2A-C catalytic activity is negatively regulated by phosphorylation at Tyr307 and C-terminal Leu309 methylation is essential for the overall phosphatase activity. The PP2A-B 56δ isoform has been reported to control Cdc25C and G2/M cell cycle checkpoint activity [29], [34]. S100A8/A9 binding to PP2A was confirmed by co-immunoprecipitation assay using antibodies against S100A8/A9 complex or PP2A. Using 27E10 antibody (specific for S100A8/A9 complex) for capture, PP2A co-immunoprecipitated with S100A8/A9 (Figure 5A). When PP2A-Aα/β antibody was used for capture, S100A9 was detected in the immunoprecipitate (marker for S100A8/A9 complex) confirming the interacting partners. S100A8/A9 expression in synchronized KB-S100A8/A9 cells (5 h post-G1/S) did not affect PP2A subunit protein levels (subunits Aα/β, B56δ and Cα/β) using immunoblotting analysis; phosphorylation of subunit Cα/β (Tyr307), however, appeared reduced (Figure 5B). C-terminal methylation appeared unaffected by S100A8/A9 expression as identified by anti-methylated-PP2A-C (Leu309) antibody.

To determine PP2A phosphatase activity, the holoenzyme from cell lysates with or without S100A8/A9 expression was isolated by immunoprecipitation using an antibody against PP2A-C catalytic subunit. The phosphatase activity of immunoprecipitated PP2A was then determined by measuring the level of inorganic phosphate (P_i_) release from a phosphopeptide substrate. For up to 7 h post-synchrony, PP2A phosphatase activity was approximately 5-fold greater in the presence of S100A8/A9 than in the absence (KB-EGFP cells), decreasing by 7-fold at 12 h post-synchrony. Phosphatase activities were normalized to detectable PP2A-Aα/β levels co-immunoprecipitated with S100A8/A9 (27E10 antibody) (Figure 5C). S100A8/A9-dependent modulation of PP2A activity in KB cells, therefore, appears to depend on the phases of the cell cycle. PP2A activity was also increased when cell lysate from KB-EGFP was preincubated with 1 µg of purified S100A8/A9 (data not shown). When KB-S100A8/A9 cells were treated with a PP2A inhibitor, okadaic acid (OA), p-Cdc25C (Ser216) was reduced to the levels of KB and KB-EGFP cells, suggesting inhibition of the S100A8/A9-mediated increase in p-Cdc25C (Ser216) phosphorylation (Figure 5D).

## Discussion

As members of the S100 family of proteins, S100A8 and S100A9 in complex (S100A8/A9 or calprotectin) are thought to have a regulatory role in cell cycle progression. In this study, we found that S100A8/A9 functions as a negative regulator of cell division and growth in KB and TR146 human carcinoma cells by inducing G2/M cell cycle arrest. KB cells were used as an in vitro model for gain-of-function study since this carcinoma line fails to express S100A8 and S100A9 mRNA and protein. TR146 is a more differentiated HNSCC cell line with detectable S100A8/A9 expression and is similar to other available lines. The ratios of S100A8 to S100A9 cannot be compared between TR146 and KB-S100A8/A9 cells, yet the attributed role in control of the G2/M checkpoint appears to be the same. Hence, if individual subunits are present in excess in either cell line, such discrepancies do not explain the purported intracellular function of S100A8/A9. From our current findings, reduced levels of S100A8/A9 and resulting loss of function de-regulates growth in SCCs and would contribute to carcinogenesis. S100A8/A9 levels are generally low or not detectable in squamous carcinoma cells, whereas in inflammatory or hyperproliferative oral lesions and HNSCC tissues, S100A8/A9 complex is markedly down-regulated at both mRNA and protein levels compared to normal mucosa [31], [32]. Consistent with these findings, we found that HNSCC show approximately 10-fold lower S100A8 and S100A9 gene expression than NAT. S100A8/A9 levels in NAT may reflect field cancerization. Although S100A8/A9 gene expression in mucosal tissues of cancer-free individuals may be even greater, decreased S100A8/A9 expression levels are clearly associated with carcinogenesis.

To determine whether the dysregulation of S100A8/A9 in HNSCC is a cause or an effect of the cancer phenotype, we sought to determine a role in cell cycle regulation. S100A8/A9 protein is found in the cytoplasm and can localize in the nucleus (The Human Protein Atlas database, http://www.proteinatlas.org) or perinuclear area [35] depending on cell growth status. During cell division and when controlling the G2/M checkpoint, S100A8/A9 is predicted to localize to the nucleus.

**Figure 6:**
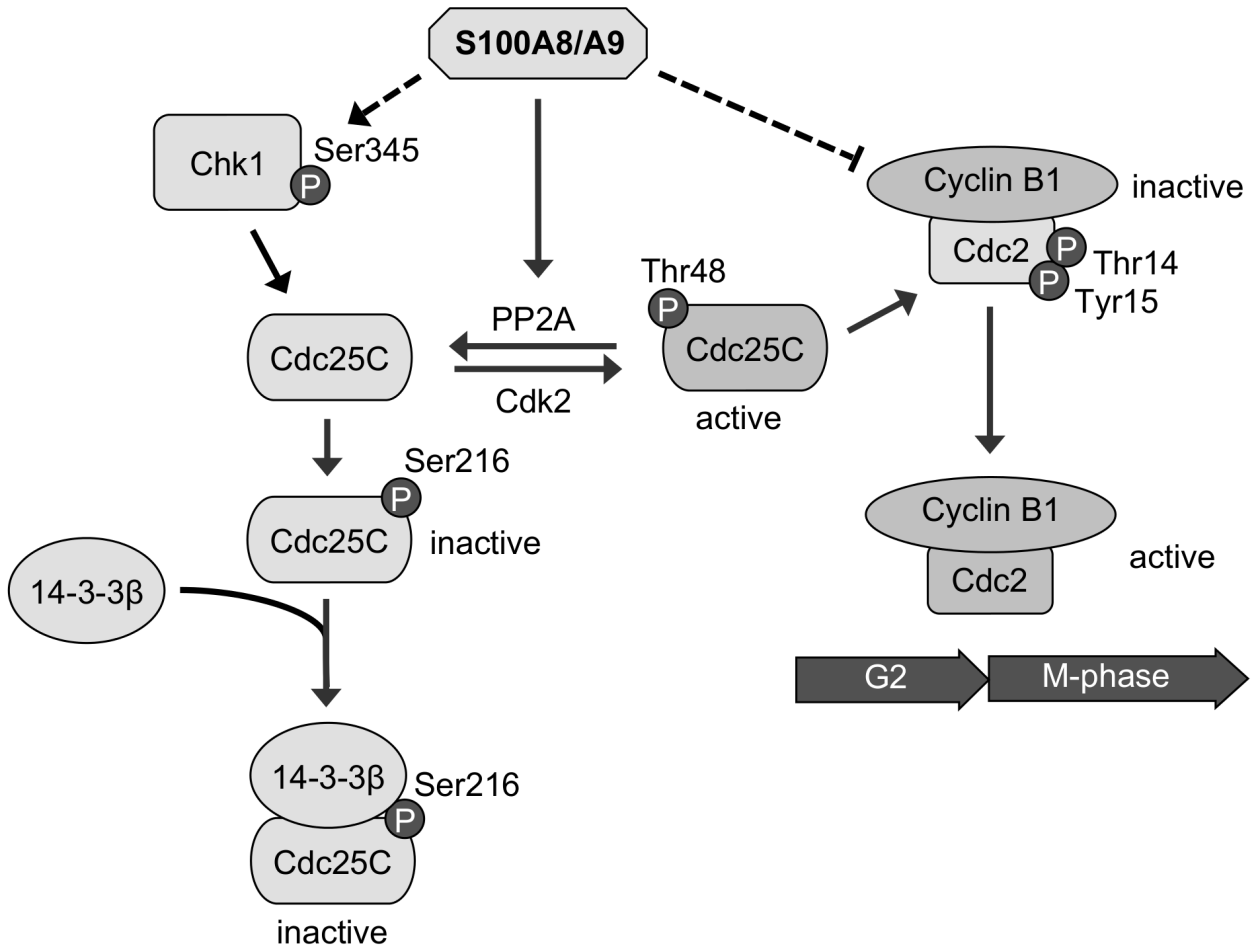
Proposed S100A8/A9 (calprotectin)-mediated regulation of the G2/M cell cycle checkpoint signaling pathway as summarized in this report. S100A8/A9 expression increases p-Chk1 (Ser345) activating phosphorylation and PP2A phosphatase activity, which is hypothesized to target and dephosphorylate p-Cdc25C (Thr48), allowing Cdc25C to be phosphorylated by p-Chk1 (Ser345) at inhibitory residue Ser216 [66]. Phosphorylated p-Cdc25C (Ser216) is targeted and bound by 14-3-3β, leading to inactivation and cytosolic accumulation of Cdc25C. As a result, cyclin B1/p-Cdc2 (Thr14/Tyr15) complex is inactivated, arresting cell cycle at the G2/M checkpoint. Solid line represents direct regulation; dashed line represents unknown mechanism.

Canonical G2/M cell cycle checkpoint signaling pathway is under the control of kinases Chk1/2 [36], [37]. Chk1/2 inactivates protein phosphatase Cdc25, which is required for activation of cyclin B/cyclin-dependent kinase 1 complex (CDK1, also known as Cdc2) and entry into mitosis [36], [37]. Activated p-Chk1 (Ser345) phosphorylates the G2/M-specific phosphatase Cdc25C at Ser216 and promotes its binding to the molecular chaperone 14-3-3β [38], [39] (see Figure 6). Binding to 14-3-3β inactivates and promotes cytosolic accumulation of p-Cdc25C (Ser216) [40]. Alternatively, active p-Cdc25C (Thr48) dephosphorylates inhibitory residues Thr14 and Tyr15 of p-Cdc2 and activates the Cdc2/cyclin B1 complex, resulting in entry into mitosis [41].

We found that S100A8/A9 increased phosphorylation of G2/M-specific kinase p-Chk1 (Ser345), which is essential for the kinase activity [42]. Ser345-hyperphosphorylation of p-Chk1 suggests that p-Chk1 (Ser345) is activated by S100A8/A9 through a mechanism that is yet unknown. S100A8/A9 did not appear to co-immunoprecipitate with Chk1 using co-immunoprecipitation (data not shown) suggesting that these proteins do not interact. We also observed marked hyperphosphorylation of p-Cdc25C (Ser216) and p-Cdc2 (Thr14/Tyr15) and increased Cdc25C/14-3-3β complex in the presence of S100A8/A9. In contrast to p-Chk1 (Ser345), phosphorylation of Cdc25C and Cdc2 at Ser216 and Thr14/Tyr15, respectively, are inhibitory. Cdc25C/14-3-3β complex formation is an indication of Cdc25C inactivation. The level of mitotically active p-Cdc25C (Thr48) and cyclin B1 was greatly reduced in the presence of S100A8/A9. Hence, S100A8/A9 is strongly suggested to activate the G2/M DNA damage checkpoint, leading to inactivation of the Cdc25C, Cdc2 and mitotic arrest. Although we were not able to characterize the activation status of Cdc25C by immunoblotting for p-Cdc25C (Thr48) in HNSCC cell lines TR146, SCC-58, OSCC-3 and UMSCC-17B, the level of p-Cdc2 (Thr14/Tyr15) hyperphosphorylation strongly correlated with the level of S100A8/A9 expression in these cells. In different HNSCC cell lines, multiple phosphorylation sites can regulate Cdc25C, including phosphorylating residues such as Thr67 or Thr130 [43]. Phosphorylation at Ser214, Ser191 and Ser198 in human Cdc25C is also possible, and Cdc25A or Cdc25B rather than Cdc25C may also be involved in the regulation of Cdc2 in different cell lines [43], [44]. In KB cells, Cdc25C appears to regulate phosphorylation of p-Cdc2 (Thr14/Tyr15), which serves as a more critical molecular marker for mitotic activity. As we show, p-Cdc2 (Thr14/Tyr15) and S100A8/A9 levels appear to correlate in multiple HNSCC cell lines.

S100A8/A9-dependent reduction in expression of cyclin B1 and hyperphosphorylation of p-Cdc2 (Thr14/Tyr15) shown in this study are consistent with Cdc2 inactivation and G2/M checkpoint arrest as commonly reported in carcinomas [45]–[47]. As confirmed in the knockdown of S100A8/A9 by shRNA in TR146 HNSCC cells, reduction in S100A8/A9 expression resulted in decreased Cdc2 phosphorylation at Thr14/Tyr15. S100A8/A9-induced inactivation Cdc2 to negatively regulate the G2/M cell cycle progression is suggested to reduce carcinoma growth and may inhibit tumor formation. Indeed, S100A8/A9-expressing KB-S100A8/A9 and TR146 cells both showed significant reduction in growth and clonogenicity when compared to S100A8/A9-negative or S100A8/A9-knockdown cells. Although KB-S100A8/A9 cells expressed less S100A8 and S100A9 than normal tissue as represented by the NAT (relative to GAPDH housekeeping gene as intracellular control), ectopic expression caused substantial growth suppression. Since knockdown of S100A8/A9 in TR146 cells resulted in marked increase in growth and colony formation in soft agar, our data, therefore, strongly suggests a concentration-dependent effect of S100A8/A9 on carcinoma cell growth and clonogenicity. Further loss of S100A8/A9 expression in tissues would be expected to accelerate tumor growth and malignant transformation in HNSCC.

Although the loss of both G1/S and G2/M checkpoints are required for increased growth and proliferation in carcinomas, S100A8/A9-dependent induction of the G2/M checkpoint contributed significantly to the suppression of mitotic activity and growth of KB cells. Expression of S100A8/A9 caused KB-S100A8/A9 cells to arrest and accumulate at G2/M, inhibiting progression into mitosis without affecting the rate of G1/S-to-G2/M transition. Overall, this growth inhibiting effect of S100A8/A9 is consistent with a report in epidermal keratinocytes, where ectopic transient expression of S100A8/A9 suppressed cell division and proliferation [48]. Further studies into the fate of the G2/M arrested carcinoma cells will be essential to explain how S100A8/A9 exerts long-term effects on cell division. Although other studies have shown concentration-dependent effects on cell growth and apoptosis by extracellular S100A8/A9 [49]–[52], so far we have not observed any changes in apoptosis in the presence or absence of S100A8/A9 expression in KB or TR146 cells (data not shown). In our experiments, the effect on carcinoma growth could not be attributed to extracellular S100A8/A9 protein since S100A8/A9 released into the medium from TR146 or transfected KB cells is below the limits of detection (data not shown) and sequence analysis of *S100A8* and *S100A9* genes shows no upstream signal peptides, which are required for canonical secretion. Hence, S100A8/A9 as studied is a cytosolic protein complex and is unlikely to be secreted by epithelium into extracellular space under normal growth conditions. S100A8/A9 expression also had no apparent effect on the levels of G1/S-associated downstream regulator proteins and the percentages of S100A8/A9-positive and -negative cells in G1/S and S phase were similar following the release from synchrony. Although a defect in G1/S has not been rigorously excluded, S100A8/A9-mediated regulation of cell cycle is strongly suggested to affect G2/M.

We now report the first mechanistic insights to explain how S100A8/A9 signals inactivation of Cdc25C and Cdc2 leading to induction of G2/M cell cycle arrest. Reduction of mitotic p-Cdc25C (Thr48) phosphoprotein is likely signaled through dephosphorylation by a Ser/Thr protein phosphatase 2A (PP2A). PP2A has been reported to induce cell cycle arrest by targeting and dephosphorylating p-Cdc25C (Thr48) [27]–[29], allowing Cdc25C to be phosphorylated at Ser216 by p-Chk1 (Ser345) for inactivation and nuclear export as described above.

The PP2A-Cα/β subunit is the enzymatic active site of PP2A and its phosphatase activity is controlled by phosphorylation at Tyr307 and methylation at Leu309, which is also essential for the holoenzyme assembly. Phosphorylation at Tyr307 of PP2A-C inhibits the phosphatase activity [53], [54]. In KB-S100A8/A9 cells, expression of S100A8/A9 did not affect the protein level of PP2A-Aα/β, -B56δ (a regulatory subunit known to signal targeting of Cdc25C) or -Cα/β subunits. Leu309 methylation levels of PP2A-C (methyl-PP2A-C) subunit were also similar in KB-S100A8/A9, KB-EGFP and wild-type control cells. These data suggest that S100A8/A9 has no regulatory effects on PP2A expression or the holoenzyme assembly in KB cells. In contrast, the phosphorylation of PP2A-C at Tyr307 was reduced in KB-S100A8/A9 cells, indicating that S100A8/A9 expression increased PP2A-C phosphatase activity.

As expected, S100A8/A9 expression augmented pre-mitotic PP2A phosphatase activity in KB-S100A8/A9 transfected cells. When purified S100A8/A9 protein was preincubated with KB-EGFP cell lysate (lacking S100A8/A9), PP2A activity was modulated similarly. Hence, S100A8/A9 appears to directly signal an increase in dephosphorylation and activation of PP2A. Post-mitotic PP2A phosphatase activity was markedly suppressed by S100A8/A9 expression, suggesting biphasic, cell cycle-dependent regulation by S100A8/A9. It should be noted that PP2A phosphatase activity was markedly elevated at time  = 0 hour post-synchrony. The level at t = 0 may reflect activity during G1/S. PP2A also plays a role in G1/S cell cycle arrest [55], [56], independent of the G2/M checkpoint signaling pathway. Elevated PP2A phosphatase activity at t = 0 was likely due to the transient G1/S blockage by aphidicolin that was used to synchronize the cells at G1/S. Although S100A8/A9 did not alter progress through G1 to S, an S100A8/A9-independent inhibition of G1/S (pharmacologically by aphidicolin in this case) could have signaled PP2A activation. Most importantly, PP2A phosphatase activity at G1/S was either dependent on or enhanced by S100A8/A9 expression in KB-S100A8/A9 cells and will be further investigated in our future studies. Interestingly, PP2A and S100A8/A9 appeared to interact directly based on co-immunoprecipitation experiments, suggesting a possible mechanism of PP2A activation by the S100A8/A9 protein complex. Additional experiments will be needed to confirm the physical interaction and the structural basis of the functional increase in phosphatase activity.

To provide further evidence that S100A8/A9-induced inactivation of Cdc25C was dependent on PP2A activity, okadaic acid treatment, which specifically inhibits PP2A activity, was shown to reduce p-Cdc25C (Ser216) phosphorylation in the presence of S100A8/A9. This finding is consistent with the literature since inactivation of Cdc25C through Ser216 phosphorylation (by Chk1) requires dephosphorylation of Cdc25C at Thr48, a known substrate for PP2A [57]. Okadaic acid treatment, therefore, inhibited S100A8/A9-induced PP2A-dependent phosphorylation of inhibitory p-Cdc25C (Ser216). Under normal growth conditions, S100A8/A9 likely mediates PP2A reactivation of the G2/M checkpoint and cell cycle delay.

In summary, we propose a model that now includes S100A8/A9 as a key signaling mediator in the induction of G2/M cell cycle checkpoint and mitotic arrest through a PP2A-dependent pathway (Figure 6). For the first time we report S100A8/A9 expression increases PP2A phosphatase activity. In carcinoma cells the G2/M cell cycle checkpoint is dysregulated, resulting in increased activation of Cdc25C and Cdc2 leading to accelerated cell growth. S100A8/A9 activates the G2/M checkpoint through PP2A to induce mitotic arrest (and reduction in growth) by increasing the levels of p-Chk1 (Ser345) activation and by inactivation of p-Cdc25C (Ser216) and p-Cdc2 (Thr14/Tyr15). Other mitotic checkpoint inhibitors are unaffected. Whether activation of Chk1 kinase (by phosphorylation at Ser345) and G2/M checkpoint is directly signaled by S100A8/A9 expression or by PP2A dephosphorylation of Cdc25C at Thr48 is still unclear, but both Thr48 and Ser216 residues of Cdc25C cannot be phosphorylated at the same time. S100A8/A9 (or calprotectin) may therefore function as a growth suppressor in carcinoma cells and reduced expression may serve as a signal for aggressive growth. Intracellular expression of S100A8/A9 in HNSCC and other SCCs could point to novel therapeutic strategies.

## Materials and Methods

### Cell lines and culture conditions

The human carcinoma cell line KB (ATCC CCL-17), negative for S100A8/A9 expression, was cultured in Minimum Essential Medium (MEM). The human HNSCC cell line TR146, SCC-58 and OSCC-3 expressing S100A8/A9 endogenously and UMSCC-17B negative for S100A8/A9 were cultured in Dulbecco's Modified Eagle's Medium/Ham's F-12 (DMEM/F-12). TR146 cells were originally derived from a cervical lymph node metastasis of a well-differentiated buccal carcinoma [58] and was a gift from Dr. Reuben Lotan, University of Texas, M.D. Anderson Cancer Center, Houston, TX USA [59]. SCC-58 and OSCC-3 originally established from primary tumor of the oral cavity [60], [61] and UMSCC-17B originally established from a metastatic neck tumor [62] were gifts from Dr. Mark Lingen (University of Chicago, Chicago, IL USA). Both MEM and DMEM/F-12 culture media were supplemented with 10% heat inactivated fetal bovine serum (complete medium) and the cells were maintained in 5% CO_2_ at 37°C. Each cell line tested *Mycoplasma* negative by qPCR before use.

### Stable expression of S100A8/A9 in human carcinoma cell line

Carcinoma cells stably transfected to express S100A8/A9 (KB-S100A8/A9, formerly known as KB-MRP8/14) or sham-control vector (KB-EGFP) were generated from KB cells as previously reported [63]. Briefly, KB cells were co-transfected with the mammalian expression vector, pIRES-EGFP (Clontech, Palo Alto, CA USA), containing *S100A8* (*MRP8*) or *S100A9* (*MRP14*) subunit genes, and the selectable marker pSV2-*neo* (G418 sulfate-resistant marker gene). The resulting cell line, KB-S100A8/A9, expresses S100A8/A9 protein complex. The KB-EGFP sham-transfection control was generated by co-transfection of insertless pIRES-EGFP and pSV2-*neo*. Both KB-S100A8/A9 and KB-EGFP were maintained in 700 µg/ml G418 sulfate (Geneticin®, Mediatech Inc., Manassas, VA USA). Cytosolic S100A8/A9 expression in transfected cells was verified both by sandwich enzyme-linked immunosorbent assay (ELISA) and indirect immunofluorescence with anti-S100A8/A9 heterodimer-specific monoclonal antibody, 27E10 (Bachem, King of Prussia, PA USA). Co-immunoprecipitation was also performed to confirm protein complex formation.

### Gene expression analysis by real-time quantitative RT-PCR

S100A8 and S100A9 expression was analyzed in clinical specimens of human head and neck squamous cell carcinoma (HNSCC) and matching NAT resected from three patients with stage II (T2N0M0), III (T3N0M0) and IVA (T4N0M0) tumors originating in the tongue, larynx and salivary gland, respectively. HNSCC tumor tissues from the three HNSCC patients and NAT, which showed no evidence of tumor cells, were obtained with informed consent through a commercial tissue bank (ProteoGenex, Inc., Culver City, CA USA). Tumors and NATs were snap frozen within 30 minutes post-resection, sectioned and analyzed for neoplastic cells. RNA was extracted from regions of the HNSCC specimens containing at least 70% tumor cells and NATs using Trizol and analyzed using an Agilent Bioanalyzer 2100 for quality control. Tumor and NAT total RNA from each patient was pooled separately, reverse transcribed, and quantified using real-time quantitative reverse-transcription polymerase chain reaction (qRT-PCR) with SYBR Green. In separate experiments, total RNA was extracted from KB and TR146 cells using a Qiagen RNeasy Mini Kit, quantified using a NanoDrop spectrophotomer, and S100A8 and S100A9-specific mRNAs were quantified using real-time qRT-PCR as above.

### Stable knockdown of S100A8/A9 in a human HNSCC cell line

To generate S100A8/A9-knockdown clones (TR146-shRNA-S100A8/A9KD) as previously described [64], TR146 cells (with endogenous S100A8/A9 expression) were transfected with the GeneEraser short hairpin RNA (shRNA) mammalian expression vector (Stratagene, Cedar Creek, TX USA) pGE-1 containing oligo sequences to produce interfering shRNA for S100A8 and S100A9 gene transcripts. Some cells were transfected with pGE-1 control vector, containing scrambled shRNA, to produce a negative control for S100A8 and S100A9 gene silenced clones (TR146-shRNA-control). Clones that grew in the presence of 250 µg/ml G418 sulfate were selected. S100A8/A9 gene and protein expression was quantified by qRT-PCR and immunoblot.

### Growth of S100A8/A9-positive and -negative cells

To determine anchorage-dependent growth rates, tumor cells were cultured on non-pyrogenic polystyrene tissue culture flasks in complete medium (see above), harvested by trypsinization and counted by trypan blue exclusion. Counts were confirmed using a Vi-Cell cell viability analyzer (Beckman Coulter, Fullerton, CA USA). Anchorage-independent growth was determined by enumerating cell colonies after growth in standard soft agar medium (0.25% agar in complete medium plated on top of 0.5% solid agar) for up to 2.5 weeks in 5% CO_2_ at 37°C.

### Cell synchronization and cell cycle analysis

KB, KB-EGFP and KB-S100A8/A9 cells were seeded at a density of 1×10^5^ cells/ml and cultured in complete medium as described earlier. To synchronize at G1/S, cells at 70% confluency (cultured for ∼48 hours) were serum starved overnight and then blocked with 3 µg/ml aphidicolin in complete medium for 12 hours. To release the G1/S block and facilitate re-entry into the cell cycle, synchronized cells were washed three times with Dulbecco's phosphate-buffered saline (DPBS) without calcium and magnesium to remove aphidicolin and cultured in fresh medium containing 10% FBS. After release from G1/S block, cells were harvested at various time points, fixed in 70% ice-cold ethanol and DNA stained at 37°C for 30 minutes with 25 µg/ml propidium iodide (PI) solution (containing 1 mg/ml RNase and 0.1% Triton X-100 in DPBS). PI-stained DNA content was analyzed using flow cytometry. Mitotic cells in synchronized cultures were also enumerated by immunostaining using phospho-histone H3 (Ser10)-specific polyclonal antibody, which was detected by PE-conjugated secondary antibody and analyzed by flow cytometry as described elsewhere [65]. To synchronize cells at G2/M, cells at 70% confluency were treated with 200 ng/ml nocodazole for 18 hours. G2/M synchronized cells were gently washed once with DPBS and harvested by trypsinization for protein extraction and analysis as described below.

### Cell lysis and protein extraction

Carcinoma cells cultured in monolayers were harvested by trypsinization, washed twice with DPBS and lysed for 2 hours on ice using immunoprecipitation lysis (IP) buffer (50 mM Tris-HCl, pH 7.5, 150 mM NaCl, 1 mM EDTA, 1 mM EGTA, 1% Triton X-100, 0.5% Nonidet P-40, 1 mM Na_3_VO_4_, 2 mM NaF) supplemented with fresh protease inhibitor cocktail (Cat# P8340, Sigma-Aldrich Biotechnology, St. Louis, MO USA) at 1∶100 dilution. Insoluble materials were removed by centrifugation at 15,000× g for 10 minutes at 4°C, the supernatants were collected, and protein concentrations determined by BCA™ protein assay (Pierce Biotechnology Inc., Rockford, IL USA).

### Immunoblot analysis

Protein samples were boiled in 1X sample buffer (1% SDS in 31.25 mM Tris-HCl, pH 6.8 with 12.5% glycerol, 0.005% bromophenol blue and 2.5% beta-mercaptoethanol) for 5 minutes. SDS-polyacrylamide gels were loaded with 50 to 100 µg total protein per well as indicated, transferred to PVDF or nitrocellulose membranes by a Bio-Rad (Bio-Rad Laboratories, Inc., Hercules, CA USA) semi-dry protein transfer apparatus, blocked overnight with 5% skim milk in TBS-T buffer (20 mM Tris-HCl pH 7.4, 137 mM NaCl, 0.1% Tween-20), and incubated with the primary antibody diluted at 1∶1000 for 1 hour in blocking buffer at room temperature. The blots were then washed three times for 5 minutes each with TBS-T and incubated for another 1 hour with horseradish peroxidase (HRP)-conjugated secondary antibody diluted at 1∶3000 in blocking buffer. All antibodies to detect cell cycle regulators were purchased from Santa Cruz Biotechnology, Inc., Santa Cruz, CA USA, except for phospho-PP2A-C (Tyr307) rabbit monoclonal antibody (Epitomics, Inc., Burlingame, CA USA). Final washes were performed three times with TBS-T before the blots were incubated in ECL Western Blotting Detection substrate (Amersham Biosciences, Piscataway, NJ USA). Chemiluminescent bands were documented and analyzed by exposing to Kodak XAR-5 X-ray film.

### Co-immunoprecipitation

An equal amount of lysate protein from each cell line was incubated with Ezview Red Protein A Affinity Gel (protein-A gel, Sigma-Aldrich) to minimize non-specific binding (pre-clear). The pre-cleared lysate was then mixed with 5 µg of capture antibody and the volume was adjusted to 1 ml with TBS. The mixture was incubated at 4°C with gentle rotation for 1 hour, followed by addition of 25 µl of protein-A gel and incubation for an additional hour at 4°C with gentle rotation. The protein-A-antibody-protein complex was then washed three times with cold immunoprecipitation lysis buffer and eluted from the protein-A gel with 1X sample buffer (as above). After brief centrifugation, the supernatants were incubated in boiling water for 5 minutes and analyzed by immunoblotting. For the 14-3-3β:Cdc25C interaction study, mouse anti-14-3-3β was used as co-IP capture antibody and rabbit anti-Cdc25C for immunoblot detection. Mouse anti-S100A8/A9 antibody 27E10 or anti-S100A9 and goat anti-PP2A-Aα antibody were used to co-immunoprecipitate S100A8/A9 and PP2A. All antibodies were purchased from Santa Cruz Biotechnology, Inc.

### PP2A inhibition by okadaic acid treatment

KB, KB-EGFP and KB-S100A8/A9 cells were seeded at a density of 3×10^5^ cells/well and cultured in complete medium overnight as described above. Cultured cells were incubated with fresh media containing 10 nM okadaic acid (Sigma-Aldrich) or vehicle control (DMSO) for 24 h. Cells were harvested by washing twice with cold DPBS and lysed directly in the well on ice with phosphatase lysis buffer (50 mM Tris-HCl, pH 7.5, 150 mM NaCl, 1% sodium deoxycholate, 1% SDS, 1% Triton X-100, 1 mM Na_3_VO_4_, 2 mM NaF and 10% protease inhibitor). Cell lysates were subjected to two freeze-thaw cycles at −80°C, centrifuged at 40°C and supernatants collected for protein quantification by BCA and immunoblotting.

### PP2A phosphatase activity assay

Phosphatase activity was performed using the Immunoprecipitation Phosphatase Assay Kit (Cat# 17-313, EMD Millipore, Billerica, MA USA). Synchronized cells were harvested, washed twice with DPBS, pelleted and resuspended in IP lysis buffer supplemented with fresh protease inhibitor cocktail as described above but without phosphatase inhibitors, Na_3_VO_4_ and NaF. Fresh cell lysates were used in each reaction. PP2A phosphatase activity was measured following the manufacturer's protocol. Briefly, 100 µg of total protein (concentration adjusted in IP lysis buffer) was mixed with 2 µg of mouse monoclonal capture antibody (PP2A, C subunit clone 1D6, EMD Millipore), isotype mouse IgG_2b_ was used as control, and the volume was adjusted to 500 µl with pNPP Ser/Thr Assay Buffer (50 mM Tris-HCl, pH 7.0, 100 µM CalCl_2_), followed by 1 h incubation at 4°C with constant rocking. After incubation, 25 µl of protein A agarose beads were added to each reaction mixture and incubation continued with constant rocking for another 1 h at 4°C. The beads were then washed three times with TBS, followed by one wash with Ser/Thr Assay Buffer and incubation in 750 µM threonine phosphopeptide (K-R-pT-I-R-R) solution diluted in Ser/Thr Assay Buffer for 10 min at 30°C with constant shaking. Total inorganic phosphate (P_i_) released was measured using Malachite Green phosphate detection solution (included in the kit).
